# *Post hoc* analysis of a randomized, double-blind, prospective trial evaluating a CXCR1/2 inhibitor in new-onset type 1 diabetes: endo-metabolic features at baseline identify a subgroup of responders

**DOI:** 10.3389/fendo.2023.1175640

**Published:** 2023-06-20

**Authors:** Valeria Sordi, Paolo Monti, Vito Lampasona, Raffaella Melzi, Silvia Pellegrini, Bart Keymeulen, Pieter Gillard, Thomas Linn, Emanuele Bosi, Ludger Rose, Paolo Pozzilli, Francesco Giorgino, Efisio Cossu, Lorenzo Piemonti

**Affiliations:** ^1^Diabetes Research Institute, IRCCS Ospedale San Raffaele, Milan, Italy; ^2^The Belgian Diabetes Registry, Academic Hospital and Diabetes Research Centre, Vrije Universiteit Brussel, Brussels, Belgium; ^3^Department of Endocrinology, University Hospitals Leuven‐Katholieke Universiteit Leuven, Leuven, Belgium; ^4^Clinical Research Unit, Medical Clinic and Polyclinic III, Center of Internal Medicine, Justus Liebig University, Giessen, Germany; ^5^Università Vita-Salute San Raffaele, Milan, Italy; ^6^Zentrum für Diabetes und Gefäßerkrankungen Münster, Munster, Germany; ^7^Department of Endocrinology and Metabolic Diseases, University Campus Bio‐Medico, Rome, Italy; ^8^Department of Emergency and Organ Transplantation, Section of Internal Medicine, Endocrinology, Andrology and Metabolic Diseases, University of Bari Aldo Moro, Bari, Italy; ^9^Department of Medical Sciences and Public Health, University of Cagliari, Cagliari, Italy

**Keywords:** IL-8, CXCR1, CXCR2, type 1 diabetes mellitus, trial, *post hoc* analyses

## Abstract

**Aim:**

In a recent randomized, multicenter trial (NCT02814838) a short-term anti-inflammatory treatment with ladarixin (LDX; an inhibitor of the CXCR1/2 chemokine receptors) did not show benefit on preserving residual beta cell function in new-onset type 1 diabetes. We present a *post hoc* analysis of trial patients in the predefined subgroup analysis developed according to baseline daily insulin requirement (DIR) tertiles.

**Method:**

A double-blind, randomized (2:1), placebo-controlled study was conducted in 45 men and 31 women (aged 18–46 years) within 100 days of the first insulin administration. Patients received LDX (400 mg twice daily) for three cycles of 14 days on/14 days off, or placebo. The primary endpoint was the area under the curve for C-peptide [AUC (0–120 min)] in response to a 2-h mixed meal tolerance test (MMTT) at week 13 ± 1. Seventy-five patients completed the week 13 MMTT and were divided into three groups according to the DIR tertiles: lower, ≤ 0.23U/kg/die (n = 25); middle, 0.24–0.40 U/kg/die (n = 24); upper, ≥ 0.41 U/kg/die (n = 26).

**Results:**

When considering the patients in the upper tertile (HIGH-DIR), C-peptide AUC (0–120 min) at 13 weeks was higher in the LDX group (n = 16) than in the placebo (n = 10) group [difference: 0.72 nmol/L (95% CI 0.9–1.34), p = 0.027]. This difference reduced over time (0.71 nmol/L at 26 weeks, p = 0.04; 0.42 nmol/L at 52 weeks, p = 0.29), while it has never been significant at any time in patients in the lower and/or middle tertile (LOW-DIR). We characterized at baseline the HIGH-DIR and found that endo-metabolic (HOMA-B, adiponectin, and glucagon-to-C-peptide ratio) and immunologic (chemokine (C-C motif) ligand 2 (CCL2)/monocyte chemoattractant protein 1 (MCP1) and Vascular Endothelial Growth Factor (VEGF)) features distinguished this group from LOW-DIR.

**Conclusion:**

While LDX did not prevent the progressive loss of beta-cell function in the majority of treated subjects, the *post hoc* analysis suggests that it could work in subjects with HIGH-DIR at baseline. As we found differences in endo-metabolic and immunologic parameters within this subgroup, this generates the hypothesis that the interactions between host factors and drug action can contribute to its efficacy. Further research is needed to evaluate this hypothesis.

## 1 Introduction

Type 1 diabetes is generally defined as a beta cell–specific T-cell‐mediated autoimmune disease (1). However, it has been suggested a potential contribution to beta cell damage mediated by an associated non‐beta cell–specific inflammatory component (2). Resident macrophage, innate lymphoid cells, natural killer cells, plasmacytoid dendritic cells, mucosal-associated invariant T cells, myeloid-derived suppressor cells, immature macrophages, and immature dendritic cells have been implicated in diabetes development either in Non-obese diabetic (NOD) mice or humans (1–5). Moreover, among innate immunity cells, a growing body of data has pointed to neutrophils as mediators of beta cell damage (6–10). With the aim to target non‐beta cell–specific inflammatory component, some randomized controlled trials targeting innate immune mediators [such as interleukin (IL)‐6R, tumor necrosis factor alpha [TNFα], and IL‐1] were developed to preserve insulin secretion in stage 3 type 1 diabetes (11–15). As neutrophil migration into inflammatory sites can be prevented by targeting CXCR1 and CXCR2 receptors, we recently tested whether blocking these chemokine receptors by an allosteric inhibitor (ladarixin, LDX) (16) was able to modify the development of type 1 diabetes. In NOD mice, LDX inhibited autoimmune insulitis and even reverted type 1 diabetes (17). Despite promising preclinical results, a phase 2 randomized study failed to demonstrate a significant effect of LDX in preserving residual beta-cell function in newly diagnosed type 1 diabetes patients (18). However, it did reveal some temporary metabolic benefits in the LDX group, particularly among patients with lower fasting C‐peptide levels and higher insulin requirements at screening. It is important to note that the complexity of the disease pathogenesis, influenced by factors such as age, environment, genetics, and disease stage, could contribute to the heterogeneous nature of innate cells and their connection to the disease (19). In this study, we present a predefined subgroup analysis focusing on the efficacy endpoints, considering the daily insulin requirement (DIR) tertiles at baseline. The findings suggest that there may be specific endo-metabolic and immunologic characteristics that can help identify either a responsive population or a particular disease stage that benefits from CXCR1/2 inhibition.

## 2 Materials and methods

### 2.1 Study design and procedures

The clinical trial was registered with ClinicalTrials.gov (NCT02814838). It was conducted in compliance with all applicable regulatory requirements. Details of patient disposition and inclusion in analysis were previously described (18). Briefly, it was a phase 2, multicenter, double blind, randomized (balancing treatment and placebo in a 2:1 fashion), placebo-controlled study in patients with new-onset type 1 diabetes to assess the efficacy and safety of LDX compared with placebo. As a minimum, inclusion criteria included age 18–45 years, new-onset (randomization within 100 days from first insulin administration) type 1 diabetes confirmed by at least one positive diabetes-related auto-antibody (anti-GAD, IAA, IA-2 antibody, and ZnT8), insulin requirement at some time and residual β-cell function as per peak stimulated (MMTT) C-peptide level >0.2 nmol/L. Exclusion criteria included patient taking pre-mixed insulin or on insulin pump, creatinine clearance <60 ml/min, ALT/AST >3 x ULN and total bilirubin >3 mg/dl, hypoalbuminemia (serum albumin < 3 g/dl), QTcF > 470 ms, and other significant comorbid conditions or administration of concomitant medications that could have biased the efficacy outcome/readout. The study was conducted in 45 men and 31 women. The patients received either LDX at a dose of 400 mg (two capsules) twice daily orally, for a total daily dose of 800 mg or an equal number of matching placebo capsules for three cycles of 14 days on/14 days off. The primary endpoint was the area under the curve (AUC) for C‐peptide in response to a 2‐h mixed meal tolerance test (AUC [0–120 min]) at week 13 ± 1. Secondary endpoints included HbA1c, daily IR, severe hypoglycemic events, and the proportion of patients achieving an HbA1c <7.0% without experience of severe hypoglycemic events.

### 2.2 Assays

C-peptide, HbA1c, glucose, glucagon, autoantibody, T-cell response *ex vivo*, and chemokine/cytokine and hormonal profile were done at a centralized laboratory. Blood samples were collected at designated time-points and centrifuged within 30 min. Cell-free serum aliquots (2 × 0.5 ml) were prepared and stored at −20°C before being shipped on dry ice within 1 month. An aliquot was retained as a reserve sample. Systemic inflammation indices were calculated by the leukocyte subgroup of complete blood count and its ratios: neutrophil/lymphocyte ratio (NLR); monocyte/lymphocyte ratio (MLR); platelet/lymphocyte ratio (PLR), and derived neutrophil/lymphocyte ratio (dNLR). Protein levels in sera were measured using the Cytokine/Chemokine, Metabolic Hormone and Adipokine Milliplex^®^ MAP Kit human magnetic bead panels (Millipore, Cat. No. HCYTOMAG-60K, HMHEMAG-34K, and HADK1MAG-61K). Samples were assayed according to the manufacturer’s instructions, and the plates were read on a Luminex xMAP instrument (Luminex Corporation). The analysis of the samples was performed with the Bio-Plex Manager 6.0 software (BioRad). Serum proinsulin was measured with Human Intact Proinsulin Elisa kit (Teco Medical Group; detection limit < 0.15 pmol/l). C-peptide was measured by a two-site immunoenzymometric assay performed on a Tosoh 600 II auto-analyzer (AIA-600 Analyte Application Manual, Tosoh Bioscience, Inc.). The estimated glomerular filtration rate (eGFR) was calculated using the Modification of Diet in Renal Disease formula. A carboxyfluorescein diacetate, succinimidyl ester (CSFE) dilution assay was used to measure the proliferative response of GAD65-and insulin-reactive CD4+ T cells. PBMC from patients were labeled with CFSE and stimulated with GAD65 (5 μg/ml) or insulin (5 μg/ml) for 7 days. HLA-A*0201 peptide dextramers (Immudex) were used to identify and enumerate CD8+ T cells (within total CD8+ cells) specific for beta-cell antigens GAD65114–123 and insulin B10-18.

### 2.3 Statistical analysis

Data are presented as mean ± standard deviation (SD) or 95% CI or median, according to their distribution. All the AUC analyses were based on actual rather than scheduled timings and were calculated using the trapezoidal rule. The 2-h C-peptide AUC after the MMTT at Week 13 ± 1 was transformed as log(x+1) values; transformed AUC was analyzed with Student’s *t*-test for unpaired data to compare the LDX and placebo group. Variables with a normal distribution were compared using unpaired Student’s *t*-test. Variables with a non-normal distribution were compared using the Mann–Whitney U test. Categorical variables were compared using the χ2 test or Fisher exact test, as appropriate. Alternative approaches were explored, including subset analysis and AUC geometric mean ratios, as described in the sections below.

## 3 Results

### 3.1 Patient disposition and predefined subgroup analysis

**Figure 1:**
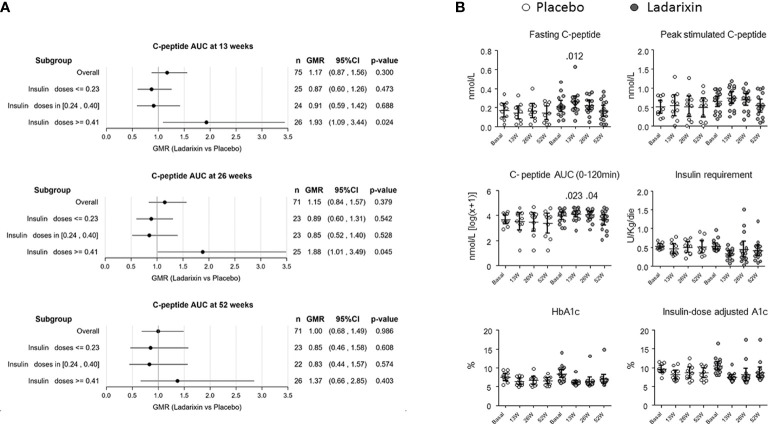
Primary and secondary outcomes in the predefined subgroup with daily insulin requirement (DIR) ≥ 0.41 U/kg/die (upper tertile). **(A)** Subgroup plot of ratios for the effect of treatment on mean area under the curve (AUC) C-peptide at 13 ± 1 (month 3), 26 ± 2 (month 6), and 52 ± 2 (month 12) weeks from the beginning of treatment. Represented are the ratio of geometric means for ladarixin (LDX) versus placebo, with 95% confidence intervals, within subgroups of patients as defined at the baseline according to the DIR tertiles: lower, ≤0.23U/kg/die (n = 25); middle, 0.24–0.40 U/kg/die (n = 24); upper, ≥ 0.41 U/kg/die (n = 26). Ab, antibody; AUC, area under the curve; GMR, geometric mean ratio. **(B)** Trial primary and secondary outcomes. Effects of ladarixin (LDX) on 2-h AUC of C-peptide AUC_(0–120 min)_, fasting C peptide, peak stimulated C-peptide, insulin requirement, HbA1c, and insulin dose–adjusted A1c. Scatter dot plots report the single patient values, and lines represent means (95% CI) for each treatment group over time. The analysis of covariance model adjusted for age, days from diagnosis, baseline HbA1c, and treatment assignment was used to compare the two groups. Only *P* values < .05 are reported in full.

Details of patient disposition and inclusion in analysis were previously described (18). In summary, a total of 73 out of 76 patients (97.3%) completed the week 52 follow-up assessment, with 48 patients receiving LDX and 25 patients receiving placebo. However, two patients receiving LDX withdrew their consent and were unable to complete the MMTT at 13 and 52 weeks, respectively. Additionally, one patient in the placebo group withdrew consent and did not complete the MMTT at 26 weeks. Therefore, the number of patients available for analysis at each time point was as follows: 76 at baseline, 75 at 13 weeks, 74 at 26 weeks, and 73 at 52 weeks. It is important to note that the primary endpoint evaluation was scheduled for the 13-week mark, and thus, 75 patients (49 on LDX, 26 on placebo) were included in the predefined subgroup analysis performed on the efficacy endpoints based on the DIR tertiles (**Figure 1A**): lower, ≤ 0.23U/kg/die (n = 25); middle, 0.24–0.40 U/kg/die (n = 24); and upper, ≥ 0.41 U/kg/die (n = 26). When considering the subgroup with DIR ≥ 0.41 U/kg/die, the 93% and 88% improvement of C-peptide AUC seen with LDX versus control at 13 and 26 weeks was nominally significant (p = 0.024 and p = 0.045, respectively, not adjusted for multiple tests), while it was not significant in the other subgroups or at weeks 52 (**Figure 1A**).

### 3.2 Baseline characteristics and efficacy outcome in HIGH-DIR and LOW-DIR

Demographic characteristics of the study groups according to the DIR tertiles are reported in **Table 1**. In agreement with the predefined subgroup analysis, “HIGH-DIR” or “LOW-DIR” was defined as those in the upper DIR tertile (≥0.41 U/kg/die, n = 26) or in the middle/lower DIR tertile (<0.41 U/kg/die, n = 49), respectively. HIGH-DIR had lower age (24.9 ± 6.5 vs. 28.4 ± 6.5 years; p = 0.032) and higher HbA1c [64 (7.9 ± 1.86) vs. 57 (7.28 ± 1.26) mmol/mol, (%); p = 0.038)] and were included earlier after diagnosis (70 vs. 77.5 days; p = 0.033). There were 16 out of 26 HIGH-DIR (61.5%) that received LDX, and there were no notable differences between treatment groups with respect to demographic and baseline characteristics (**Table 2**). MMTT-stimulated C-peptide AUC_(0–120 min)_ (adjusted for age, days from diagnosis, and baseline HbA1c) was different between the treatment groups at 13 weeks (LDX 4.17 nmol/L, 95% CI 3.8–4.54; placebo 3.44 nmol/L, 95% CI 2.9–3.9; p = 0.023) and 26 weeks (LDX 4.1 nmol/L, 95% CI 3.7–4.5; placebo 3.4 nmol/L, 95% CI 2.9–3.9; p = 0.04; **Figure 1B**). Concordantly, the results of the linear mixed model for the AUC_(0–120 min)_ throughout the study adjusted for the same factors showed a statistically significant effects for the treatment (p = 0.025). Fasting C-peptide (**Figure 1B**) was different between the treatment groups at 13 weeks (LDX 0.261 nmol/L, 95% CI 0.2–0.32; placebo 0.135 nmol/L, 95% CI 0.06–0.21; p = 0.012) and the linear mixed model confirmed a statistically significant effect for the treatment throughout the study (p = 0.041). Peak stimulated C-peptide, insulin requirement, HbA1c, and insulin dose–adjusted A1c were not statistically different throughout the study between LDX and placebo (**Figure 1B**). As expected, among the efficacy outcomes, none was different between LDX and placebo in LOW-DIR (**Supplementary Figure 1**).

**Table 1:** Characteristics of the study groups.

	LOW-DIR (N = 49)	HIGH-DIR (N = 26)	p
Age (years)			
Mean	28.4 ± 6.5	24.9 ± 6.5	0.032
Median	27	23.5	
Range	18–46	18–41	
Male sex [N (%)]	29 (59.2)	16 (61.5)	1
Ethnic group [N (%)]			0.33
White/Caucasian	49 (100)	25 (96.2)	
No. of autoantibodies [N (%)]			
1	8 (16.3)	3 (11.5)	0.79
2	16 (32.7)	10 (38.5)	
3	12 (24.5)	8 (30.8)	
4	13 (26.5)	5 (19.2)	
IAA+	21 (42.9)	11 (42.3)	1
GADA+	46 (94)	23 (88.5)	0.41
IA-2A+	29 (59.2)	15 (57.7)	1
ZnT8A+	33 (67.3)	18 (69.2)	1
No. of days from first insulin to treatment			
Median	77.5	70	0.033
Range^§^	29–107	34–100	
Weight (kg) Range	68.3 44–110.4	66.9 47.2–94.4	0.89
BMI Range	22.8 18.4–34.5	22.6 18.2–30.8	0.74
White blood cells (cells/mm^3^)	5.87 ± 1.58	5.76 ± 1.17	0.59
Neutrophil (cells/mm^3^)	3.32 ± 1.25	3.17 ± 0.94	0.41
Lymphocyte (cells/mm^3^)	1.9 ± 0.48	1.92 ± 0.46	0.79
Platelet	232,425 ± 58,534	241,500 ± 59,263	0.63
Neutrophil–lymphocyte ratio (NLR)	1.8 ± 0.69	1.73 ± 0.65	0.45
Platelet–lymphocyte ratio (PLR)	129 ± 44	128 ± 39	0.98
Lymphocyte–monocyte ratio (LMR)	5.19 ± 1.7	4.6 ± 1.9	0.28
Creatinine (μmol/L)	70.2 ± 12.2	68.1 ± 12.1	0.73
Creatinine clearance (ml/min)*	129 ± 35	135 ± 30	0.45
Fasting C-peptide (nmol/L)	0.23 ± 0.13	0.19 ± 0.1	0.168
Peak stimulates C-peptide (nmol/L)	0.71 ± 0.27	0.59 ± 0.27	0.073
C-peptide AUC (0–120) (nmol/L)	63 ± 25	53 ± 25	0.084
HbA_1c_ (mmol/mol, (%))	57 (7.28 ± 1.26)	64 (7.9 ± 1.86)	0.038
HbA_1c_ ≥7% [N (%)]	25 (52.1)	17 (65.4)	0.33
Insulin requirement (U/kg/day)	0.22 ± 0.11	0.53 ± 0.13	<0.001
Insulin-dose adjusted A1c (IDAA1c)^ç^	8.2 ± 1.5	10.2 ± 1.9	<0.001
IDAA1c ≥9% [N (%)]	14 (29.2)	19 (73.1)	<0.001

All are means ± SD, unless otherwise specified.

§: Two patients in the LOW-DIR group were randomized slightly after 100 days from the first insulin injection (day 103 and day 106); exemption was granted due to patients being already committed to study participation. Such a delay was not considered to impact the trial outcome.

*: Cockcroft–Gault formula.

^ç^ calculated as “A1c (%) + 4x insulin dose (units per kilogram per 24 h).

**Table 2:** Characteristics of the HIGH-DIR group according to the treatment.

	LDX (N = 16)	Placebo (N = 10)	
Age (years)			
Mean	25.6 ± 7.21	23.8 ± 5.43	0.652
Median	23.5	23	
Range	18-41	18-35	
Male sex [N (%)]	9 (56.3)	7 (70)	0.68
Ethnic group [N (%)]			
White/Caucasian	15 (93.8)	10 (100)	1
No. of autoantibodies [N (%)]			
1	2 (12.5)	1 (10)	0.407
2	8 (50)	2 (20)	
3	4 (25)	4 (40)	
4	2 (12.5)	3 (30)	
IAA+	6 (37.5)	5 (50)	0.689
GADA+	14 (87.5)	9 (90)	1
IA-2A+	7 (43.8)	8 (80)	0.11
ZnT8+	11 (68.8)	7 (70)	1
No. of days from first insulin to treatment			
Median	62.5	73.5	0.162
Range	34–100	47–99	
Weight (kg) Range	65.8 47.2–91.4	70.15 61.7–94.4	0.246
BMI Range	22.1 18.2–29.4	22.7 19.9–30.8	0.268
White blood cells (cells/mm^3^)	6.08 ± 1.15	5.24 ± 1.04	0.074
Neutrophil (cells/mm^3^)	3.37 ± 0.86	2.84 ± 1.02	0.169
Lymphocyte (cells/mm^3^)	2 ± 0.53	1.79 ± 0.30	0.257
Platelet	228,100 ± 64,897	228,100 ± 64,897	0.59
Neutrophil–lymphocyte ratio (NLR)	1.77 ± 0.56	1.66 ± 0.80	0.69
Platelet–lymphocyte ratio (PLR)	125 ± 33	132 ± 50	0.65
Lymphocyte–monocyte ratio (LMR)	4.52 ± 1.5	4.84 ± 2.5	0.69
Creatinine (μmol/L)	70.5 ± 14.1	67.18 ± 12.3	0.55
Creatinine clearance (mL/min)*	126 ± 26	149 ± 31	0.057
Fasting C-peptide (nmol/L)	0.21 ± 0.11	0.17 ± 0.1	0.34
Peak stimulates C-peptide (nmol/L)	0.65 ± 0.27	0.5 ± 0.25	0.18
C-peptide AUC_(0-120)_ (nmol/L)	58.6 ± 25.7	43.21 ± 20	0.124
HbA_1c_ (mmol/mol, (%))	68 (8.39 ± 2.1)	60 (7.54 ± 1.33)	0.267
HbA_1c_ ≥7% [N (%)]	11 (68.8)	6 (60)	0.692
Insulin requirement (U/kg/day)	0.53 ± 0.16	0.53 ± 0.08	0.92
Insulin-dose adjusted A1c (IDAA1c)^ç^	10.5 ± 2.1	9.66 ± 1.33	0.278
IDAA1c ≥9% [N (%)]	12 (75)	7 (70)	1

All are means ± SD, unless otherwise specified.

*: Cockcroft–Gault formula.

### 3.3 Immunologic and endo-metabolic features in HIGH-DIR and LOW-DIR

**Figure 2:**
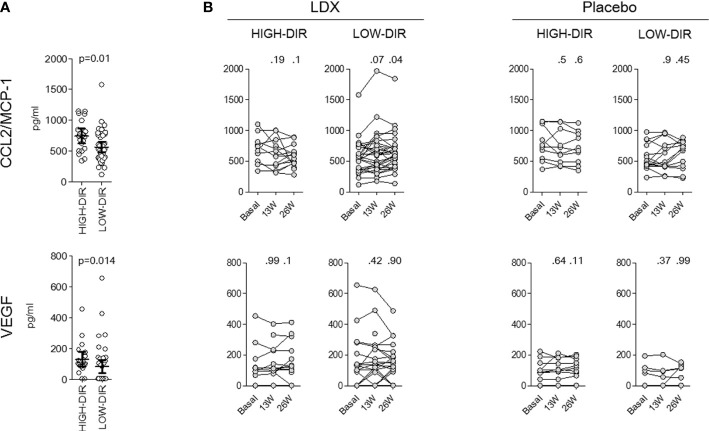
Circulating CCL2/MCP-1 and VEGF levels in HIGH-DIR and LOW-DIR before and after LDX treatment. In agreement with the predefined subgroup analysis, “HIGH-DIR” or “LOW-DIR” was defined as those in the upper DIR tertile (≥0.41 U/kg/die, n = 26) or in the middle/lower DIR tertile (<0.41 U/kg/die, n = 49), respectively. **(A)** Baseline circulating CCL2/MCP-1 and VEGF. Scatter dot plots report the single patient values, and lines represent means (95% CI). The Mann–Whitney U test was used to compare differences between the two groups. **(B)** Circulating CCL2/MCP-1 and VEGF levels before, during, and after LDX/placebo treatment in HIGH-DIR and LOW-DIR. Scatter dot plots report the single patient values, and lines connect the values of the same patient during the time. The Wilcoxon signed-rank test was used to compare between baseline and subsequent time points (13W and 16W). All P values vs. baseline are reported.

**Figure 3:**
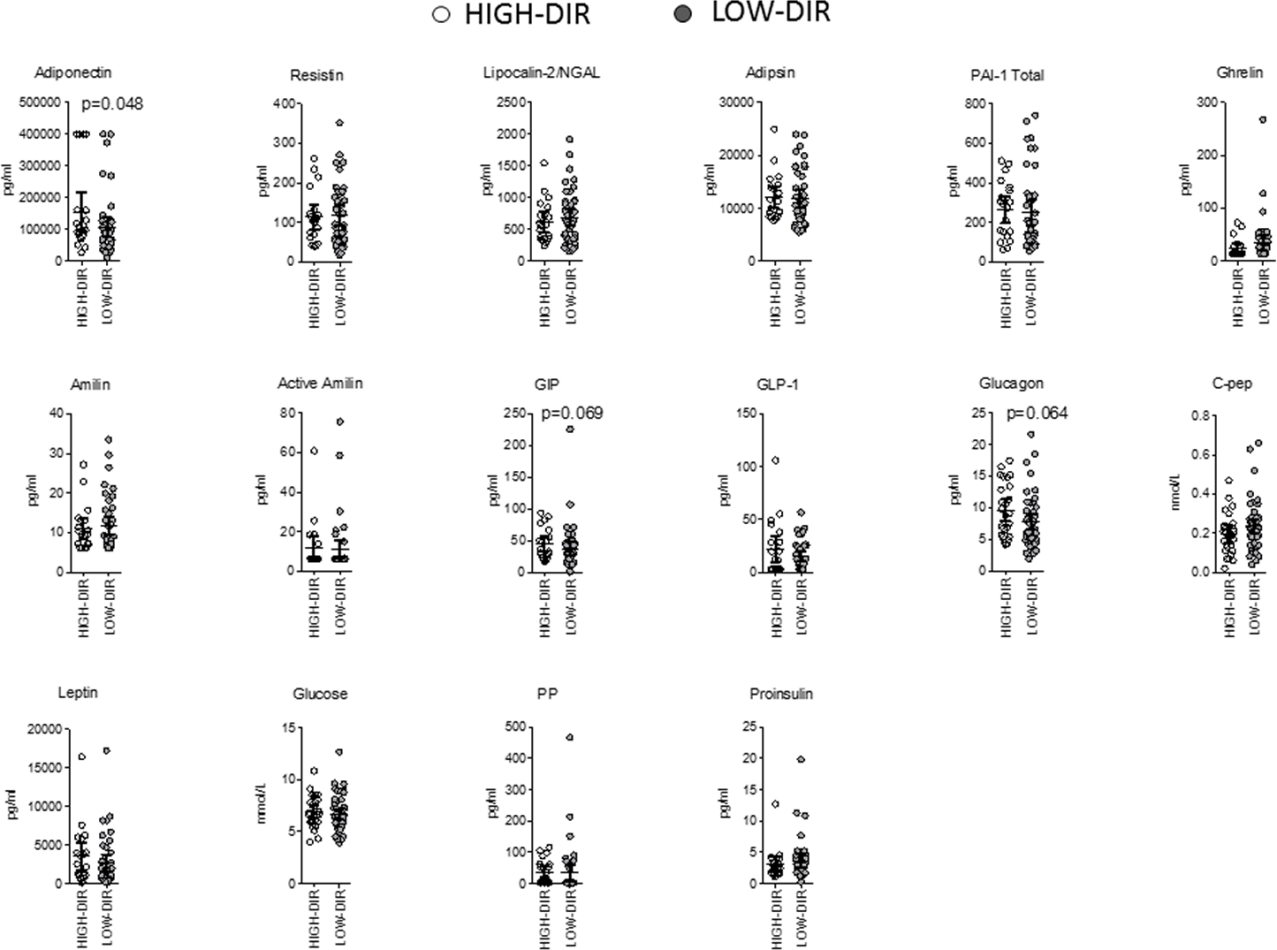
Baseline circulating adipokines and hormones in HIGH-DIR and LOW-DIR. In agreement with the predefined subgroup analysis, “HIGH-DIR” or “LOW-DIR” was defined as those in the upper DIR tertile (≥ 0.41 U/kg/die, n = 26) or in the middle/lower DIR tertile (<0.41 U/kg/die, n = 49), respectively. Scatter dot plots report the single patient values and lines represent means (95% CI). The Mann–Whitney U test was used to compare differences between the two groups. Only P values < .1 are reported in full.

**Figure 4:**
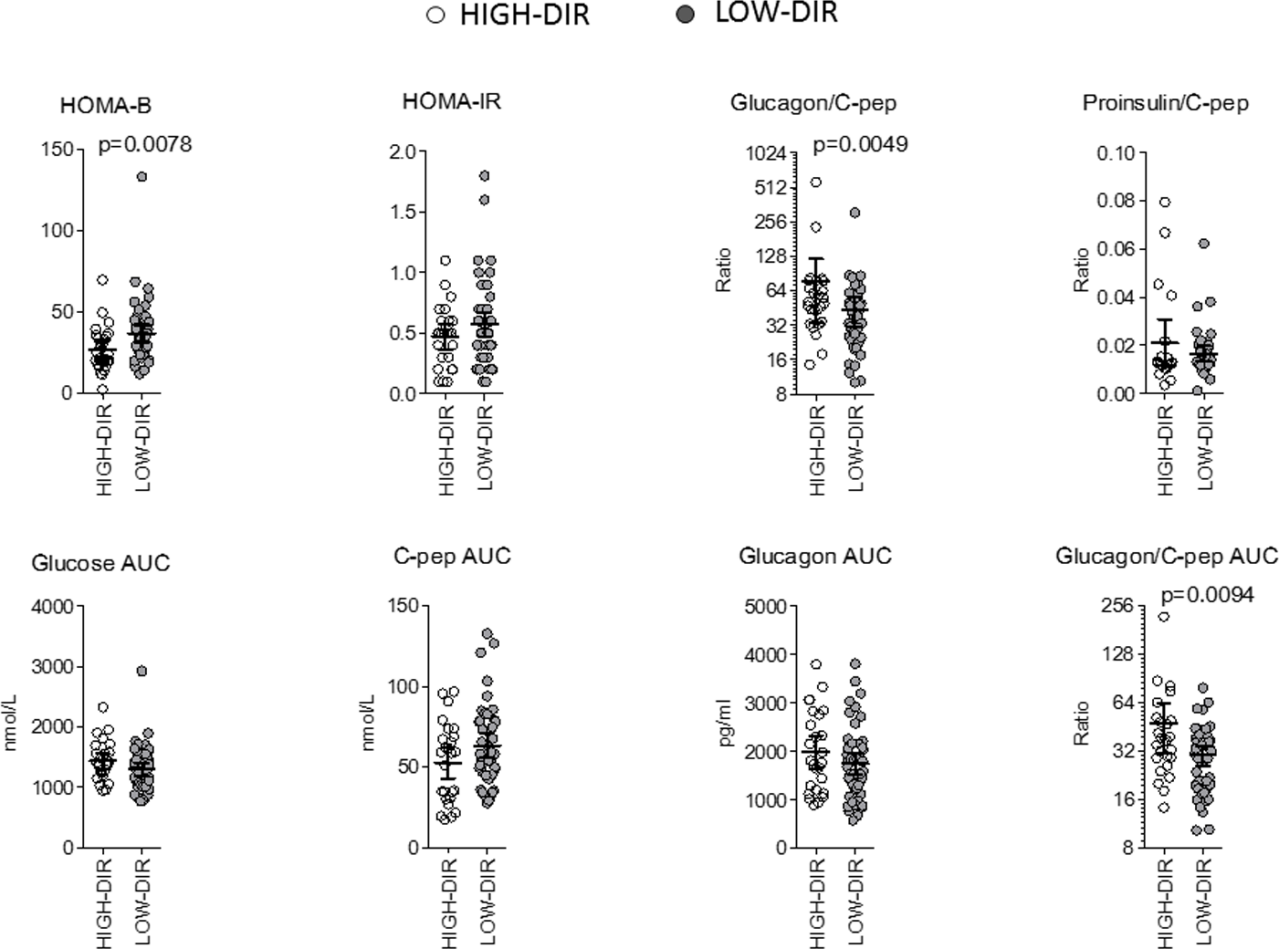
Parameters recorded during baseline mixed meal tolerance test in HIGH-DIR and LOW-DIR. In agreement with the predefined subgroup analysis, “HIGH-DIR” or “LOW-DIR” were defined as those in the upper DIR tertile (≥0.41 U/kg/die, n = 26) or in the middle/lower DIR tertile (<0.41 U/kg/die, n = 49), respectively. Scatter dot plots report the single patient values, and lines represent means (95% CI). The Mann–Whitney U test was used to compare differences between the two groups. Only P values < .1 are reported in full.

*Post hoc* analysis was performed to identify immunological and metabolic variables that differentiated at baseline HIGH-DIR (n = 26) from the LOW-DIR (n = 49). Baseline autoantibody titers (IAA, GADA IA2A, and ZnT8), circulating autoreactive T cells (GAD65-and insulin-responsive CD4 or CD8 T cells) and inflammatory indices derived from blood cell counts (dNLR, NLR, MLR, and PLR) were not statistically different between the two groups (**Supplementary Figure 2**). Among circulating cytokines and chemokines, baseline levels of CCL2/MCP-1 and VEGF were higher in HIGH-DIR than in LOW-DIR (**Figure 2A** and **Supplementary Figure 3**). Of note, in patients treated with LDX, circulating levels of CCL2/MCP-1 slightly decreased during the time in HIGH-DIR, while they significantly increased in LOW-DIR (**Figure 2B**). No changes over time were evident in placebo-treated patients (**Figure 2B**). Among circulating adipokines and hormones, baseline levels of adiponectin were higher in HIGH-DIR than in LOW-DIR (**Figure 3**). Moreover, a trend toward increased glucagon and GIP levels in HIGH-DIR was evident (**Figure 3**). To better characterize the endo-metabolic features, the *post hoc* analysis was also performed on parameters recorded during baseline MMTT (**Figure 4**). As expected, β-cell function estimated by homeostatic model assessment (HOMA-B) was lower in HIGH-DIR than in LOW-DIR. Of note, the glucagon-to-C-peptide ratio (both fasting and during MMTT) was higher in HIGH-DIR than in LOW-DIR.

## 4 Discussion

This study describes a *post hoc* analysis of a randomized controlled trial of LDX in patients with newly diagnosed type 1 diabetes. The analysis focused on the HIGH-DIR and LOW-DIR in the trial, identified by their daily insulin requirement at baseline. The HIGH-DIR were found to be younger, with higher HbA1c levels and lower HOMA-B and included earlier after diagnosis. The study also looked at immunological and metabolic features, with higher levels of CCL2/MCP-1 and VEGF in HIGH-DIR and higher levels of adiponectin, glucagon, and GIP. The glucagon-to-C-peptide ratio was also found to be higher in HIGH-DIR. These results suggest that HIGH-DIR may have a different underlying pathophysiology and could potentially benefit more from therapies like LDX. Some of the factors that have been previously identified as predictors of better response to type 1 diabetes treatment include younger age at onset, higher levels of C-peptide (a marker of insulin production), lower HbA1c levels (indicating better long-term glucose control), absence of autoantibodies (suggesting a milder form of the disease), and fewer symptoms at diagnosis (20–22). Other factors that have been associated with poorer response include delayed diagnosis, longer duration of symptoms, higher initial blood glucose levels, and presence of certain genetic variants (23–27). However, it is important to note that these factors are not absolute predictors of treatment response, and there is considerable variability among individuals with type 1 diabetes and different treatment.

Overall, our result suggests that early diagnosis and prompt treatment initiation may improve outcomes in type 1 diabetes treated with LDX, particularly in patients with β-cell dysfunction, which is indicated by higher HbA1c and DIR and lower HOMA-B and C-peptide levels. In our trial, β-cell dysfunction may suggest that the immune system is still actively attacking the insulin-producing cells in the pancreas, making prevention therapies potentially more effective in stopping further damage. This hypothesis is also supported by previous research that found a greater response to teplizumab among individuals with lower C-peptide responses to a glucose tolerance test (28). C-peptide is a marker of insulin production, and a lower response could indicate that the immune system may still be active in attacking pancreatic β cells. Therefore, individuals with a low β-cell function at onset may benefit more from prevention therapies, as their immune system may be more susceptible to intervention.

The other differences in endo-metabolic and immunologic features between HIGH-DIR and LOW-DIR to LDX in type 1 diabetes further suggest the presence of different degrees of β-cell dysfunction and immune dysregulation.

The glucagon-to-C-peptide ratio is a significant marker of alpha cell dysfunction in the pancreas. In individuals with type 1 diabetes, this ratio is often increased due to the damage and loss of beta cells. This leads to reduced insulin secretion and elevated glucagon levels, resulting in an imbalance in hormone secretion and an elevated glucagon-to-C-peptide ratio. Normally, alpha cells secrete glucagon, which raises blood glucose levels by stimulating glycogenolysis and gluconeogenesis. In healthy individuals, the release of insulin from beta cells helps inhibit glucagon secretion and maintain glucose homeostasis. However, in type 1 diabetes, the loss of beta cell function leads to inadequate insulin production and reduced inhibitory signals on alpha cells. As a result, glucagon secretion remains unchecked, causing an elevated glucagon-to-C-peptide ratio. Additionally, insulin resistance, which primarily affects peripheral tissues, can also impact alpha cell function and glucagon secretion. In individuals with insulin resistance, alpha cells may become less responsive to the inhibitory effects of insulin (29). This can lead to increased glucagon secretion and a higher glucagon-to-C-peptide ratio, further exacerbating hyperglycemia.

Adiponectin is a hormone secreted by adipose tissue that plays a role in regulating glucose and lipid metabolism. In autoimmune type 1 diabetes, adiponectin levels have been shown to be elevated at the time of diagnosis (30–32). Adiponectin levels in newly diagnosed autoimmune type 1 diabetes were reported to be positively associated with higher HbA1c levels, lower serum C-peptide, high degree of weight loss before diagnosis, the presence of diabetic ketoacidosis, higher degree of metabolic decompensation, and clinical indices of catabolism (30–32). Overall, these findings suggest that elevated adiponectin levels at the time of diagnosis of autoimmune type 1 diabetes may be indicative of more severe disease and poorer glycemic control.

CCL2/MCP-1 is a chemokine produced by various cell types and acts as a chemoattractant for monocytes, T cells, and natural killer cells, involved in recruiting immune cells to sites of inflammation. Its levels are elevated in patients with type 1 diabetes, particularly during the early stages of the disease (33, 34), contributing to the inflammatory response that leads to the destruction of insulin-producing β cells in the pancreas (35). The increase in CCL2 may identify a more immunologically active phase of the disease, making it potentially more susceptible to anti-inflammatory strategies.

VEGF is a potent angiogenic factor that plays a key role in the development and maintenance of blood vessels in the body. In type 1 diabetes, increased VEGF expression has been observed in various tissues, including the retina and kidney, and is thought to be involved in the pathogenesis of microvascular complications. The presence of VEGF in individuals with type 1 diabetes at onset was positively associated with indicators of glycemic control and inflammatory parameters (Th1, Th1/Th2 ratio), while being negatively associated with the percentage of Treg cells (36–38). As for CCL2/MCP-1, the increase in VEGF may identify a more immunologically active phase of the disease, making it potentially more susceptible to anti-inflammatory strategies.

Our study has some limitations. The first limitation is the small number of participants. The second limitation is that, even if started by a predefined subgroup analysis, the study remains *post hoc* analysis. While *post hoc* analysis can be useful for generating new hypotheses and exploring potential mechanisms of action, it is important to note that the results of such analyses are exploratory in nature and should be interpreted with caution. As such, *post hoc* analyses should be viewed as preliminary and hypothesis-generating, rather than definitive or conclusive (39). The third limitation is that no correction for multiple testing was applied. This strategy was chosen as the analysis was exploratory in nature, aware that it can inflate the probability that a Type I error (false discovery) will occur. The fourth limitation is related to the original study design, which included a short duration of treatment and a limited range of age within the studied population.

In summary, this *post hoc* analysis suggests the existence of a clinical condition (either a population or a disease stage) responsive to IL-8 inhibition characterized by a higher degree of β-cell dysfunction (higher HbA1c and DIR and lower HOMA-B and C-peptide) and identified by higher levels of CCL2/MCP-1, VEGF, glucagon-to-C-peptide ratio, and adiponectin. These findings suggest that baseline levels of certain cytokines, adipokines, and endocrine hormones may be useful in predicting response to LDX treatment in patients with type 1 diabetes. It is important to note that these findings are preliminary and further research is needed to fully understand the role of innate immune cells and IL-8 receptors in type 1 diabetes. Additionally, while the LDX group showed some transient metabolic benefits in certain subgroups of patients, it is unclear whether this would translate into long-term improvements in disease management or overall health outcomes. Nonetheless, these results provide valuable insights into the complex interplay between the immune system and metabolism in type 1 diabetes and may inform future treatment strategies for this challenging disease.

## Data availability statement

The raw data supporting the conclusions of this article will be made available by the authors, without undue reservation.

## Ethics statement

The studies involving human participants were reviewed and approved by Comitato Etico dell’IRCCS San Raffaele. The patients/participants provided their written informed consent to participate in this study.

## Author contributions

VS: conceptualization and methodology; PM: conceptualization and methodology; VL: conceptualization and methodology; RM: investigation; SP: investigation; BK: investigation, methodology, and review and editing; PG: investigation and review and editing; TL: investigation and review and editing; EB: investigation and review and editing. LR: investigation and review and editing. PP: investigation and review and editing; FG: investigation and review and editing; EC: investigation and review and editing; LP: conceptualization, methodology, formal analysis, and writing (original draft). LP is the guarantor of this work and, as such, had full access to all the data presented in the study and takes responsibility for their integrity and for the accuracy of data analysis. All authors contributed to the article and approved the submitted version.
