# Supplementary material for: Post hoc analysis of a randomized, double-blind, prospective trial evaluating a CXCR1/2 inhibitor in new-onset type 1 diabetes: endo-metabolic features at baseline identify a subgroup of responders
Source: Front Endocrinol (Lausanne). 2023 Jun 20;14:1175640. doi: 10.3389/fendo.2023.1175640 (PMC10319139; doi:10.3389/fendo.2023.1175640)
Supplement: Supplementary file 1 [file DataSheet_1.docx]

Supplementary Material

**Post-hoc analysis of a randomized, double blind, prospective trial evaluating a CXCR1/2 inhibitor in new-onset type 1 diabetes: endo-metabolic features at baseline identify a subgroup of responders.**

Sordi Valeria, Paolo Monti, Vito Lampasona, Raffaella Melzi, Silvia Pellegrini, Bart Keymeulen, Pieter Gillard, Thomas Linn, Emanuele Bosi, Ludger Rose, Paolo Pozzilli, Francesco Giorgino, Efisio Cossu, Lorenzo Piemonti *

# * Correspondence: Lorenzo Piemonti [piemonti.lorenzo@hsr.it](mailto:piemonti.lorenzo@hsr.it)

**
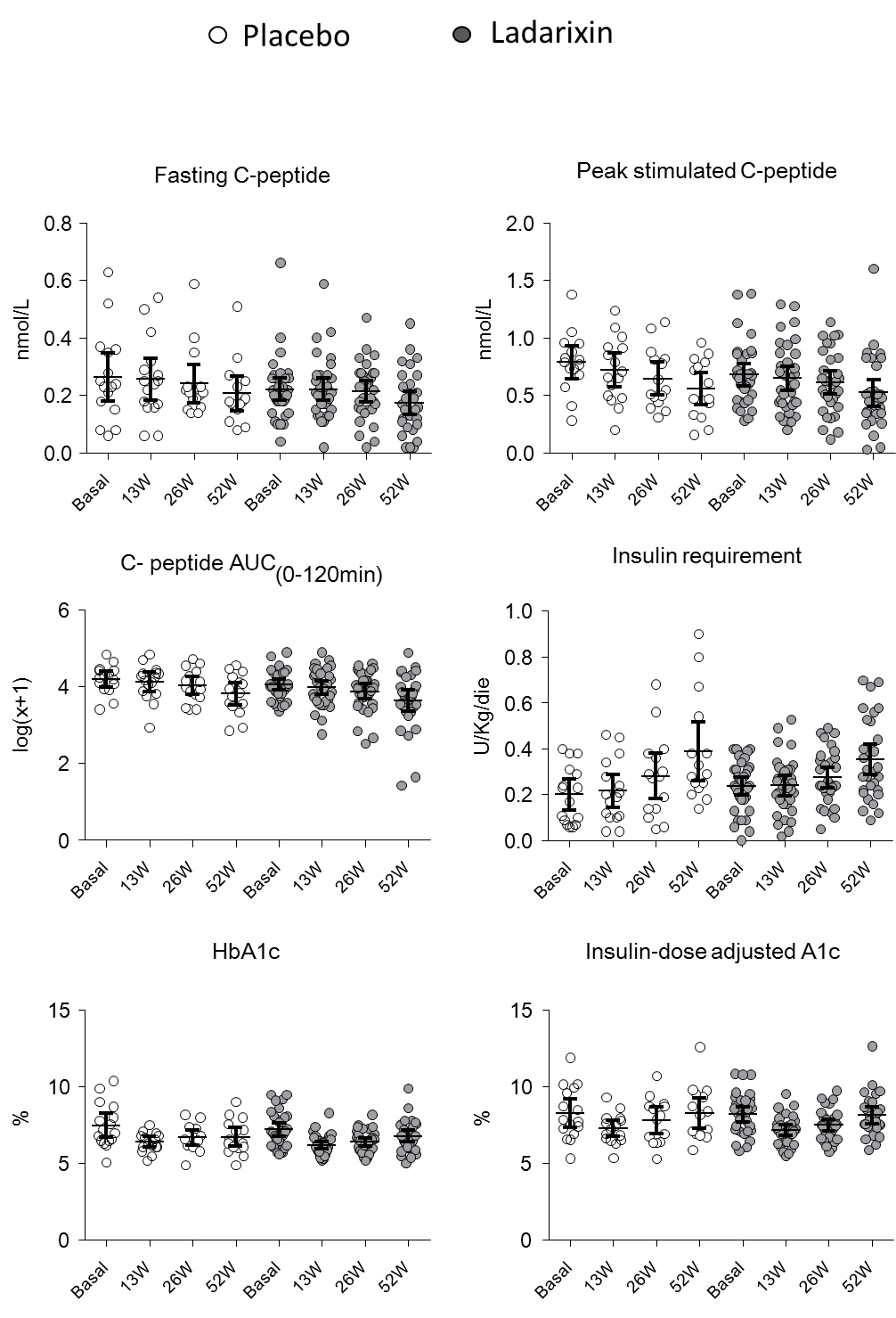
**

**Supplementary figure 1.** Primary and secondary outcomes in the predefined subgroup with DIR less than 0.41 U/kg/die (upper tertile). Effects of ladarixin (LDX) on 2-hour area under the curve (AUC) of C-peptide AUC(0-120 min), fasting C peptide, peak stimulated C-peptide, insulin requirement, HbA1c and insulin dose adjusted A1c. Scatter dot plots report the single patient values and lines represent means (95% CI) for each treatment group over time. The analysis of covariance model adjusted for adjusted for age, days from diagnosis, baseline HbA1c and treatment assignment was used to compare the two groups. P values < .05, if any, are reported in full.

**
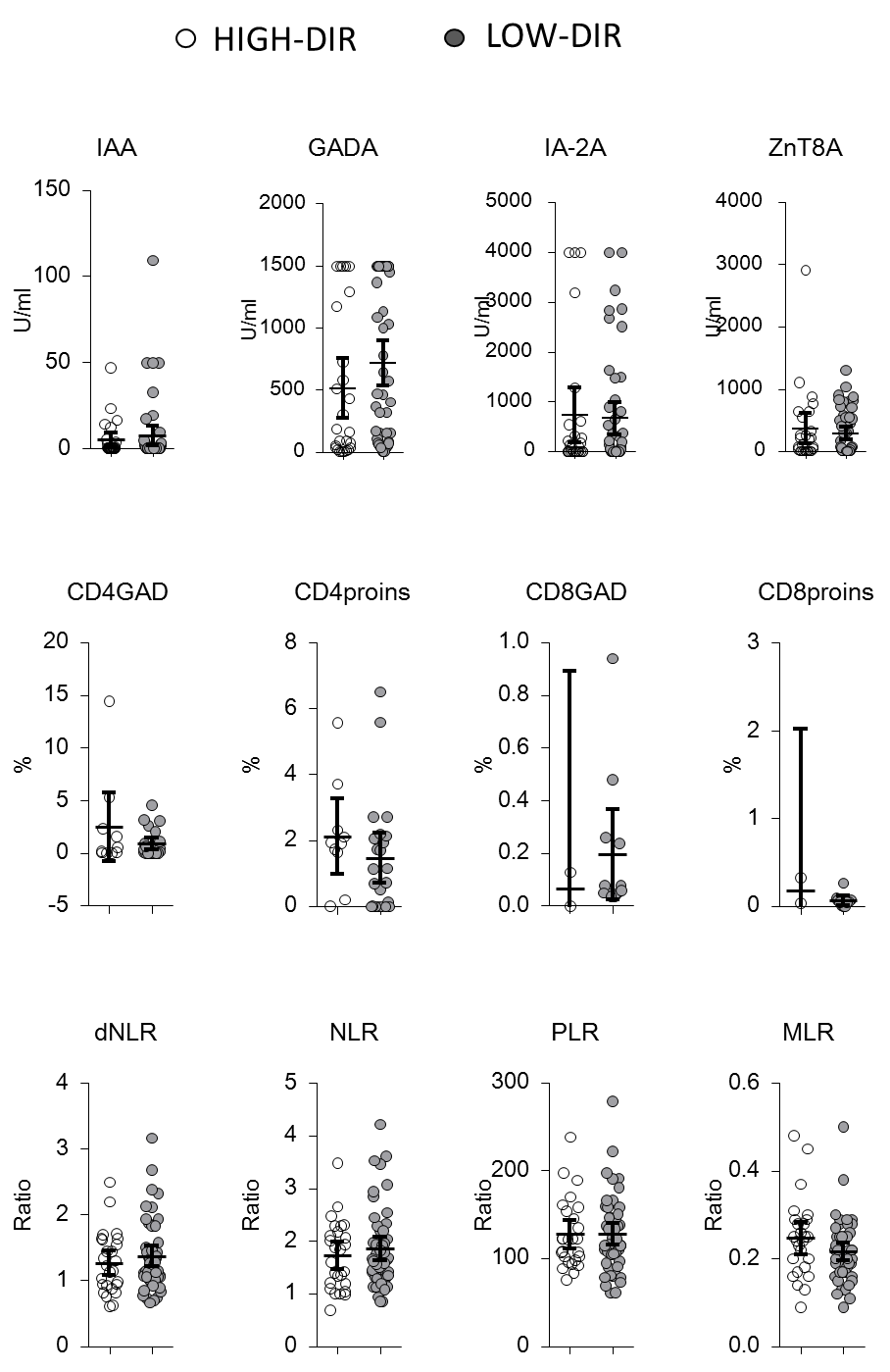
**

**Supplementary figure 2.** Baseline immunological signature in HIGH-DIR and LOW-DIR. In agreement with the predefined subgroup analysis, “HIGH-DIR” or “LOW-DIR” were defined as those in the upper DIR tertile (≥ 0.41 U/kg/die, n=26) or in the middle/lower DIR tertile (< 0.41 U/kg/die, n=49), respectively. Upper panels: baseline autoantibody titers to insulin (IAA), to Glutamic Acid Decarboxylase (GADA), to Insulinoma associated antigen 2 (IA-2A) to Zinc Transporter 8 (ZnT8A). Middle panels: baseline circulating autoreactive T cells. A CSFE dilution assay was used to detect GAD65-and insulin-responsive CD4 T cells. PBMC from patients (10 out of 26 HIGH-DIR, 23 out of 46 LOW-DIR) were labeled with CFSE and stimulated with GAD65 (5 μg/ml) or insulin (5 μg/ml) for 7 days. The graphs show the proportion of CD4^+^CFSEdimT cells that have proliferated to Ags. We measured CD8^+^T cells (within total CD8^+^ cells) specific for β cell antigens GAD65_114–123_ and insulin B10-18 identified by positive staining for HLA-A*0201 peptide dextramers in HIGH-DIR (n=2) and LOW-DIR (n=12), as this analyses can be performed only in individuals with HLA-A*0201. Lower panels: systemic inflammation indices including neutrophil/lymphocyte ratio (NLR), monocyte/lymphocyte ratio (MLR), platelet/lymphocyte ratio (PLR), derived neutrophil/lymphocyte ratio [dNLR; neutrophils/(white blood cells - neutrophils)].

**
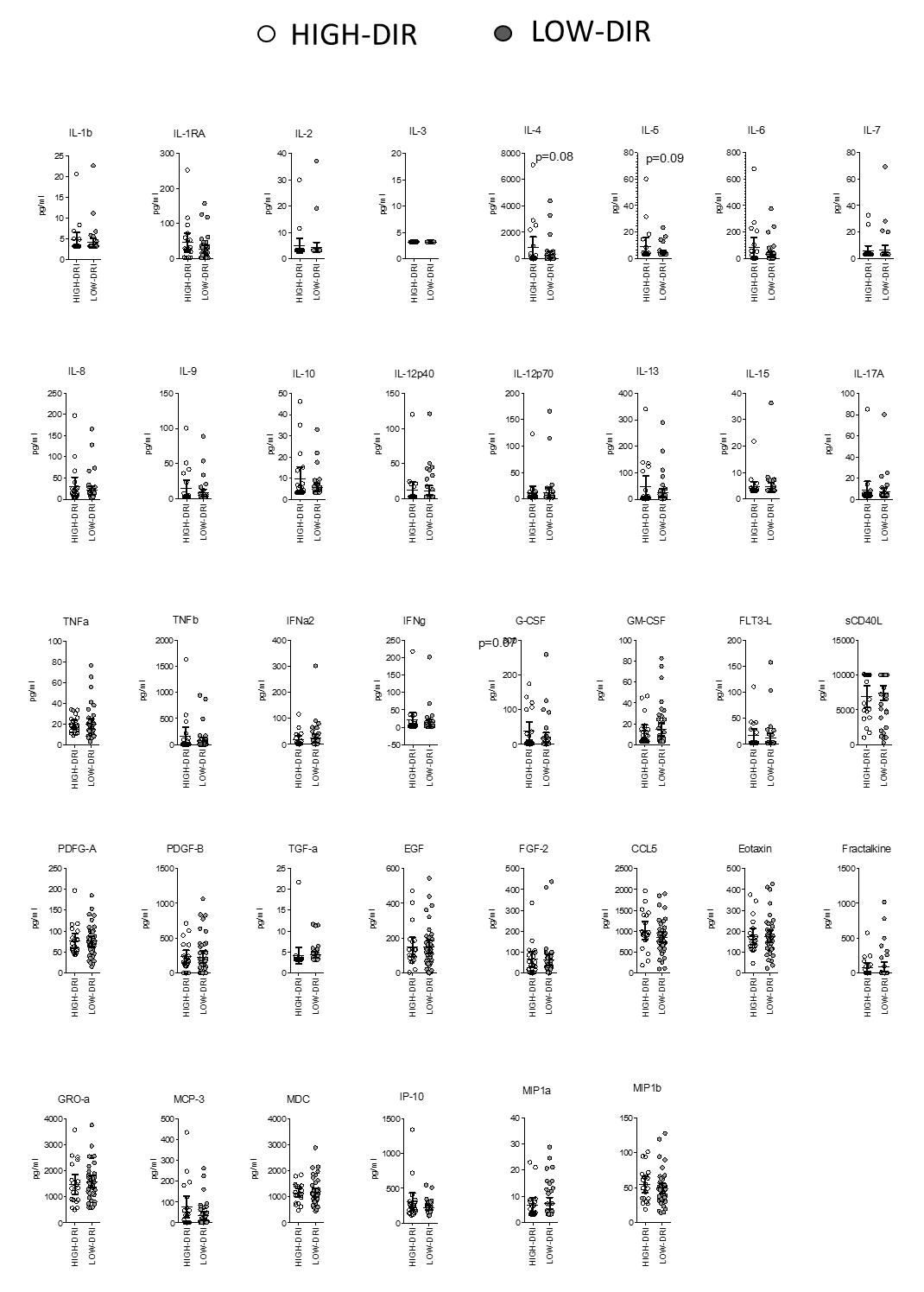
**

**Supplementary figure 3. Baseline circulating cytokines and chemokines in HIGH-DIR and LOW-DIR**. In agreement with the predefined subgroup analysis, “HIGH-DIR” or “LOW-DIR” were defined as those in the upper DIR tertile (≥ 0.41 U/kg/die, n=26) or in the middle/lower DIR tertile (< 0.41 U/kg/die, n=49), respectively. Scatter dot plots report the single patient values and lines represent means (95% CI). The Mann-Whitney U test was used to compare differences between the two groups. Only P values < .1 are reported in full.
